# JNMA from 2005-2024: Progress over Two Decades

**DOI:** 10.31729/jnma.8475

**Published:** 2024-02-29

**Authors:** Prajwala Yogi, Saroj GC

**Affiliations:** 1 Managing Editor, Journal of Nepal Medical Association, Siddhi Sadan, Kathmandu, Nepal; 2 Deputy Managing Editor, Journal of Nepal Medical Association, Siddhi Sadan, Kathmandu, Nepal

Dr Achala Vaidya was appointed chief editor for the Journal of Nepal Medical Association (JNMA) by Dr Sudha Sharma, President of Nepal Medical Association, in 2005.^1^ Then, the Chief Editor appointed Dr Angel Magar as Deputy Executive and Web Editor to index the JNMA after recognizing his potential to help the Kathmandu University Medical Journal index into PubMed.^2^ The new era of JNMA began with various transformations over the years. Prof Dr Anjani Kumar Jha, then President of NMA (2013),^3^ appointed Dr Magar as Editor-in-Chief for the JNMA.^1^ The Journal has achieved many milestones during this time, such as digitising all printed copies of the JNMA since 1963, with support from the WHO SEARO, New Delhi, India.^4^ The Journal started publishing bimonthly,^5^ then monthly in 2020.^6^ The student JNMA section was also introduced in the same year.^7^ JNMA also benefited from Dr Magar's international associations with the Council of Science Editors (CSE),^8^ the Committee on Publication Ethics (COPE),9 the International Society of Managing and Technical Editors (ISMTE),^10^ the World Association of Medical Editors (WAME)^11^ and the Asia Pacific Association of Medical Journal Editors (APAME).^12^

Apart from the publication of the Journal, Dr Magar also focused on improving academic medicine in Nepal by conducting various trainings, workshops, and seminars. Some of them include but are not limited to JNMA Leadership Programme, Scientific Writing and Publishing Articles in Dentistry, JNMA Training on Research and Publication for DM/MCh, JNMA Research grant, and How to Prepare and Work Efficiently during Government Bond Posting in Nepal? Some of these programs were conducted outside Kathmandu Valley in Chitwan, Birgunj, Dharan, Palpa, Bhairahawa, Biratnagar, Nepalgunj, etc. The main aim of the training was to increase awareness and educate using a bottom-up approach by reaching medical students, young medical graduates, and early career doctors and conducting thought-provoking discussions with a top-down approach. Undoubtedly Dr Angel has been an inspiration and mentor for many medical students, young graduates and researchers including us who are seeking guidance on personal development, career opportunities, research and publication. There are over 200 programmes by JNMA during this period, some of these were:-

**1. JNMA elective:** This program is for young medical graduates, intern medical doctors, and medical students (on vacation/awaiting exam results) to provide them with insight into the research and publication environment for a month. During the elective, they were given the necessary training on research and publication to work effectively according to the JNMA system. The earliest electives were started back in 20062007 with more than 18 programs to date, each with 10 to 30 participants, and a duration of 30 days.

**2. JNMA Training on Scientific Writing:**^13^ The foremost JNMA training was on scientific writing since 2008. JNMA has conducted over 18 programs, with each program accommodating between 25 and 65 participants and having a duration of one day. From delving into the nuances of scientific writing to navigating the peer review and publication processes, participants gained invaluable insights and practical skills essential for scientific publication. With a comprehensive curriculum and hands-on workshop covering study design and all aspects of manuscript preparation, submission, and publication, attendees were empowered to contribute significantly to advancing science and medical education.

**3. JNMA Hands-On Training on EndNote:** The EndNote training on reference management software was to streamline creating, organizing, and formatting references within research documents with the help of software since 2009. The program empowers academics, post-graduate residents, and research-focused individuals, equipping them with essential skills for navigating reference management complexities and ensuring accurate, formatted citations. JNMA has held over 6 workshops, each with a participant range of 17 to 25 and a duration of one day.

**4. Advanced Techniques for Scientific Literature Search:** It was designed to address the challenges and uncertainties faced by medical professionals in systematic and practical approaches to conducting a literature search for research purposes. With a focus on the art of literature searching, participants have gained insights into effective techniques, locating full-text articles, and managing references, along with an introduction to systematic review. This program introduced in 2010, has organized more than 6 programs, hosting between 10 to 30 participants in each.

**5. JNMA Workshop on Hinari Basic Course:** It focused on accessing free journals available through HINARI, since 2010.

**6. beyond Medical School:** It is tailored for medical students and offers insights into academic medicine, emphasizing research, publication, and career importance. It covers postgraduation aspects such as career opportunities, finance management, speciality planning, professionalism, marriage, and personal growth, facilitating a holistic understanding of life beyond medical education. This initiative commenced in 2013 and has organized more than 18 programs, each with a participant count ranging between 50 and 120 and lasting for one day.

**7. beyond INTERNSHIP:** It is designed for a medical intern doctor and young medical graduate and offers insights into shaping their career, navigating personal milestones, managing family expectations and emphasizing research and publication since 2009. JNMA has organised over 16 programs, each attended by 50 to 80 participants and having a duration of one day. Participants acquire a comprehensive understanding of medical research, the scientific publication process, and the significance of evidence-based medicine. It covers post-graduation aspects such as career opportunities, finance management, speciality planning, professionalism, marriage, and personal growth, facilitating a holistic understanding of life beyond the internship.

**8. JNMA Golden Jubilee Anniversary- The 50 Glorious Years of JNMA:**^14^ JNMA organized its 50th anniversary, recognizing the contribution of the past chief editors along with past presidents for their valuable contribution to establishing the JNMA as one of the leading journals of Nepal in 2013.

**9. JNMA Short Course for Medical Journal Editors:** It was a 10-week intensive short course for Medical Journal Editors conducted in 2017. It also supported the establishment of a new medical journal in Nepal by the Nepal Police Hospital.

**10. Designing and Conducting a Clinical Research (DCCR):** The research is conducted through a scientific process, unlike without any systematic approaches. To improve the way research is being carried out, this program was conducted under various names such as Short Course, Basic Training, Principle and Practice, and A Primer on DCCR, which was conducted over a day and over four and six weeks as its extended version since 2017. The program, which has been conducted over 1 to 30 days, has benefited between 20 to 60 participants in each program, totalling more than 15 programs.

**11. JNMA Hands-on Training for Fast Track Publication:** This unique training emphasizes hands-on preparation of scientific articles, specifically tailored for authors submitting or planning to submit articles to the JNMA since 2017. It includes fast-track review and peer review processes, ensuring timely communication and potential acceptance within four weeks of submission. JNMA has conducted over 44 programs, with each program hosting between 15 to 35 participants and lasting for one day.

**12. Introduction to Research and Scientific Publication:** It aims to enhance local science by empowering medical students, young medical graduates, and early career medical doctors by introducing them to research and publications since 2017.

**13. Medical Research Made Easy:** This training program has been designed to equip doctors, interns, medical students, and aspiring researchers with the essential knowledge and skills needed to conduct scientific research since 2017. The training aims to empower attendees to initiate and pursue research projects confidently by providing practical insights and structured guidance.

**14. JNMA Hands-On Training on Thesis Writing:** This program has been designed for MD/MS and later extended to DM/MCh residents to support their research works by organizing a hands-on training program on thesis writing since 2013. It provides in-depth scientific knowledge on conducting a thesis, offering practical insights and strategies.

**15. Introduction to Medical Statistics:** Medical Statistics is a separate specialty. However, it has not received the recognition and attention it should have in our country. This programme aims to introduce statistics to the researcher, and research focus individuals since 2018.

**16. JNMA Short Course on Peer Review:** Peer review is an integral part of journal publication. To increase the pool of peer reviewers, this programme has been conducted since 2013.

**17. Critical Appraisal Course:** It is designed for doctors, dentists, nurses, health professionals, and post-graduate students; this program imparts critical appraisal skills to evaluate clinical research papers since 2019. Participants learn to identify study designs, assess methodologies, and confidently judge research paper usefulness. The goal is to foster evidence-based practices, empowering individuals to contribute to evidence-based medicine.

**18. Webinars during COVID-19:** During the COVID-19 period we conducted webinars such as NMA PAN NHTC TeleECHO™ on Psychological First Aid, Stress Management, and Grief Counselling, NMA Webinar on Psychological First Aid, Stress Management, and Grief Counselling, NMA WHO MoHP program on COVID-19 for intern doctors, medical students, and dental students, NMA WHO NHTC Critical Care Training for healthcare workers, and Stress Management in COVID-19 for Frontline health workers in Nepal. Around 11,000 healthcare workers were trained on how to care for critically ill COVID-19 patients.^15-18^

**19. Women in Medicine:**^19^ This programme recognizes the contribution of Nepalese Women in Medicine since 2021. It was organized and kept under the calendar event of NMA, acknowledging women who have made significant contributions to the medical field, whether through groundbreaking research, innovative clinical practices, or leadership roles.

**20. National Health Summit:**^20-22^ JNMA Editor-in-Chief and then NMA President Dr Lochan Karki brainstormed on organizing a new health summit with the primary objective of establishing a distinguished platform for healthcare professionals, experts, and stakeholders to convene, collaborate, and participate in the programme. The first summit was organized in 2017, aligning with the JNMA anniversary in September each year.

**21. Past President Forum:** The concept of a "Past President Forum" is a valuable and strategic initiative that involves bringing together individuals who have previously served as presidents of an organization, association, or institution since 2022. The forum serves as a platform for collaboration, knowledge-sharing, and sharing experiences with future generations. It also aligned with a leadership programme.

**22. The JNMA Research Placement:** It is a unique opportunity for young medical graduates, intern doctors, and medical students to immerse themselves in the world of scientific research and publication within the context of a medical journal since 2023. By joining JNMA, participants walk through the entire journal workflow, act as goodwill ambassadors, and receive training on research and publication in the JNMA format.

Dr Angel's journey's highlights have been the notable increase in a testament to high-quality research contributions from our authors and the rigorous peer-review process upheld by our dedicated team and reviewers. The Journal has played a pivotal role in maintaining academic rigour and integrity. According to Editor-in-Chief Dr Angel Magar, the collaboration and logistics support of the President, general secretary, other executive members of NMA, and technical support from NMA staff, especially Chief Administrative Officer Mr. Milan Chandra Khanal, has been an asset to the Journal. Dr Magar also stated that NMA leadership has been instrumental in the Journal's progress, particularly from Dr Kedar Narsingh KC, NMA President (2009-2011), and Prof Dr Choplal Bhusal, NMA President (2007-2009). Prof Dr Anjani Kumar Jha, NMA President (2013-2016), Dr Muktiram Shrestha, NMA President (2009-2011). Dr Lochan Karki, NMA President (2020-2023) and Dr Sudha Sharma, NMA President (2005-2007).^3^
